# Tulobuterol patch alleviates allergic asthmic inflammation by blockade of Syk and NF-κB activation in mice

**DOI:** 10.18632/oncotarget.24348

**Published:** 2018-01-31

**Authors:** Lixia Fu, Jing Guan, Yujia Zhang, Pei Ma, Yuanyuan Zhuang, Jinye Bai, Yasi Ding, Qi Hou, Wan Gong, Mingbao Lin, Wensheng Zheng, Jianmin Zhang

**Affiliations:** ^1^ State Key Laboratory of Bioactive Substances and Functions of Natural Medicines, Institute of Materia Medica, Chinese Academy of Medical Sciences and Peking Union Medical College, Beijing, China; ^2^ Beijing Shouer Pharmaceutical Factory, Capital Institute of Pediatrics, Beijing, China; ^3^ College of Basic Medical Science, Zhejiang Chinese Medical University, Hangzhou, China

**Keywords:** tulobuterol patch, allergic airway inflammation, mouse, Syk, NF-κB

## Abstract

**Background:**

Tulobuterol patch, one of strongest bronchodilators, was recently shown to improve bronchial hyperresponsiveness and significantly decrease the sputum eosinophil counts by combining with nonspecific anti-inflammatory drugs on patients with asthma. However, there is limited study on the anti-inflammatory activities of tulobuterol patch and its potential machenism.

**Results:**

The tulobuterol patch significantly ameliorated inflammatory cell infiltration in the lung tissue, reduced the number of total leukocytes and its differential count, markedly reduced the production of IL-1β, TNF-α, IL-6, CCL-11 and IL-4 in bronchial alveolar lavage fluid, as well as a reduction in IL-4/IFN-γ ratio. Tulobuterol patch exhibited the best effect on allergic inflammation compared with formoterol and salbutamol. Furthermore, tulobuterol patch treatment significantly suppressed the expression and activation of Syk and its downdream signaling NF-κB and p-NF-κB.

**Conclusions:**

The present studies revealed that tulobuterol patch effectively ameliorated airway inflammatory responses in allergic asthma, and its mechanisms, at least partially, via down-regulating Syk/NF-κB pathway.

**Methods:**

An ovalbumin induced allergic asthma mouse model were used, and the effects of tulobuterol patch on allergic airway inflammation were evaluated. Also, its anti-airway inflammatory potential was compared with two other β_2_-agonists, salbutamol and formoterol. Its possible anti-inflammatory mechanisms were identified by using western blotting and immunohistochemistry.

## INTRODUCTION

Allergic asthma is a heterogeneous inflammatory lung disease affecting millions of people worldwide and with a steadily increasing incidence [1]. Asthma treatments are predominantly the combination of nonspecific anti-inflammatory drugs (inhaled corticosteroids, ICS) and bronchodilators (β_2_-agonists), which work in most patients [2]. However, the use of ICS has been associated with growth impairment in children and other systemic adverse effects, such as an increased risk of pneumonia, hyperglycemia, hypertension, osteopenia to patients with large dosages and/or long-term treatment [3, 4]. This leads to poor adherence to ICS and increases the risk of asthma exacerbations. These pitfalls call for some alternative or auxiliary anti-asthma drugs with no or lower toxicity [4], especially for children.

Tulobuterol is a short-acting selective β_2_-agonist. The tulobuterol patch containing molecular and crystallized forms of tulobuterol provides a favorable pharmacokinetic profile and avoids adverse drug reactions, which make it a useful long-acting β_2_-agonist with good adherence [5–7]. Recently, some β_2_-agonists are shown to attenuate the proinflammatory activities of a range of immune and inflammatory cells *in vitro*, such as neutrophils, monocytes, mast cells, eosinophils, basophils, and lymphocytes, all of which contribute to the pathogenesis of various acute and chronic respiratory diseases [8]. Recent clinical studies also showed that tulobuterol patch as an add-on medication decreased the sputum eosinophil count more significantly compared with ICS or leukotriene receptor antagonist treated alone on patients with asthma [5, 6]. However, the anti-inflammatory activities of tulobuterol patch and its potential machenism is of considerable potential value in the pharmacotherapy of allergic asthma with limited study.

In allergic asthma, airway inflammation is characterized as a T helper (Th) 2 lymphoyte immune response to allergens, by hyper-production of allergen-specific IgE, which binds to high-affinity IgE receptor (FcϵRI) of mast cells and eosinophils, followed by degranulation and release of multiple cytokines [9]. The spleen tyrosine kinase (Syk) plays a critical role in FcεRI-dependent inflammation in inflammatory cells, and triggers a complex series of signaling pathways, including the activation of the nuclear factor-κB (NF-κB), producing a vast array of inflammatory mediators [10, 11]. Considering that syk is involved in the proximal part of signalling pathways initiated by allergen-mediated activation of immunoreceptors on inflammatory cells [12], it may represent an attractive target for new therapeutics.

Therefore, in this study, we investigated the potential value of tulobuterol patch in the pharmacotherapy of allergic airway inflammation by using an ovalbumins (OVA)-induced allergic airway inflammation mouse model, and identified its possible anti-inflammatory mechanism by down-regulating of syk and NF-κB.

## RESULTS

### Tulobuterol patch decreased the levels of inflammatory cells in bronchial alveolar lavage fluid (BALF)

**Figure 1:**
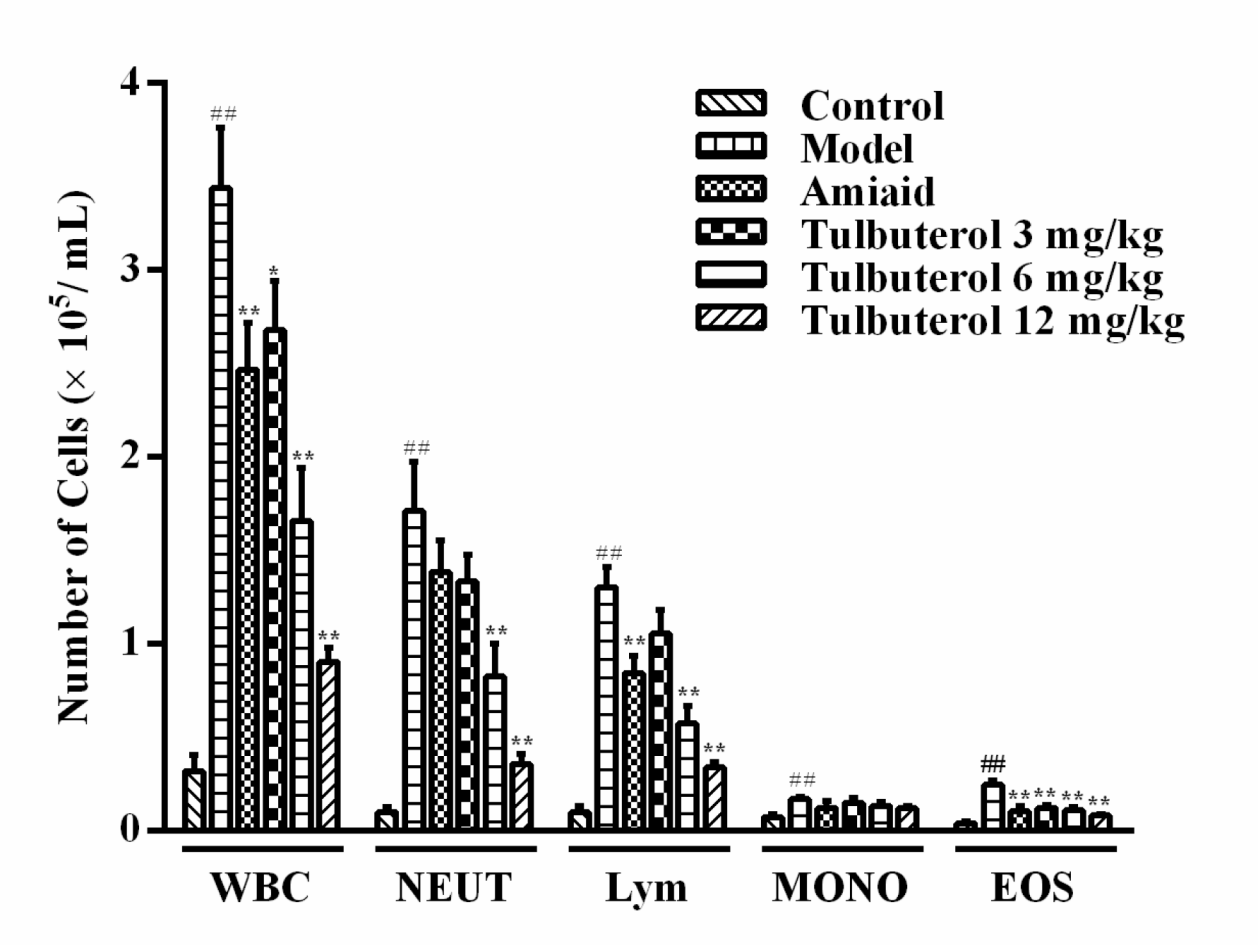
The recruitment of total leukocytes and and its differential count in BALF Data were expressed as mean ± SD (*n* = 10), WBC = total leukocytes, NEUT = neutrophils, Lym = lymphocytes, MONO = monocytes and EOS = eosinophils. ^#^*P* < 0.05 and ^##^*P* < 0.01 *vs.* control group; ^*^P < 0.05 and ^**^P < 0.01 *vs.* model group.

To investigate the effects of tulobuterol patch on the recruitment of inflammatory cells, total numbers of leukocytes and its differential count in BALF were determined. As shown in Figure 1, compared with the control group, sensitization and challenge with OVA resulted in a markedly increase in the total leukocytes, neutrophils, lymphocytes, monocytes and eosinophils counts in BALF (model group). Tulobuterol patch treatment remarkably decreased the counts of total leukocytes, neutrophils, lymphocytes, eosinophils in a dose-independent fashion, compared to the model group (*p* < 0.05 or *p* < 0.01).

### Tulobuterol patch decreased the levels of inflammatory mediators in BALF

**Figure 2:**
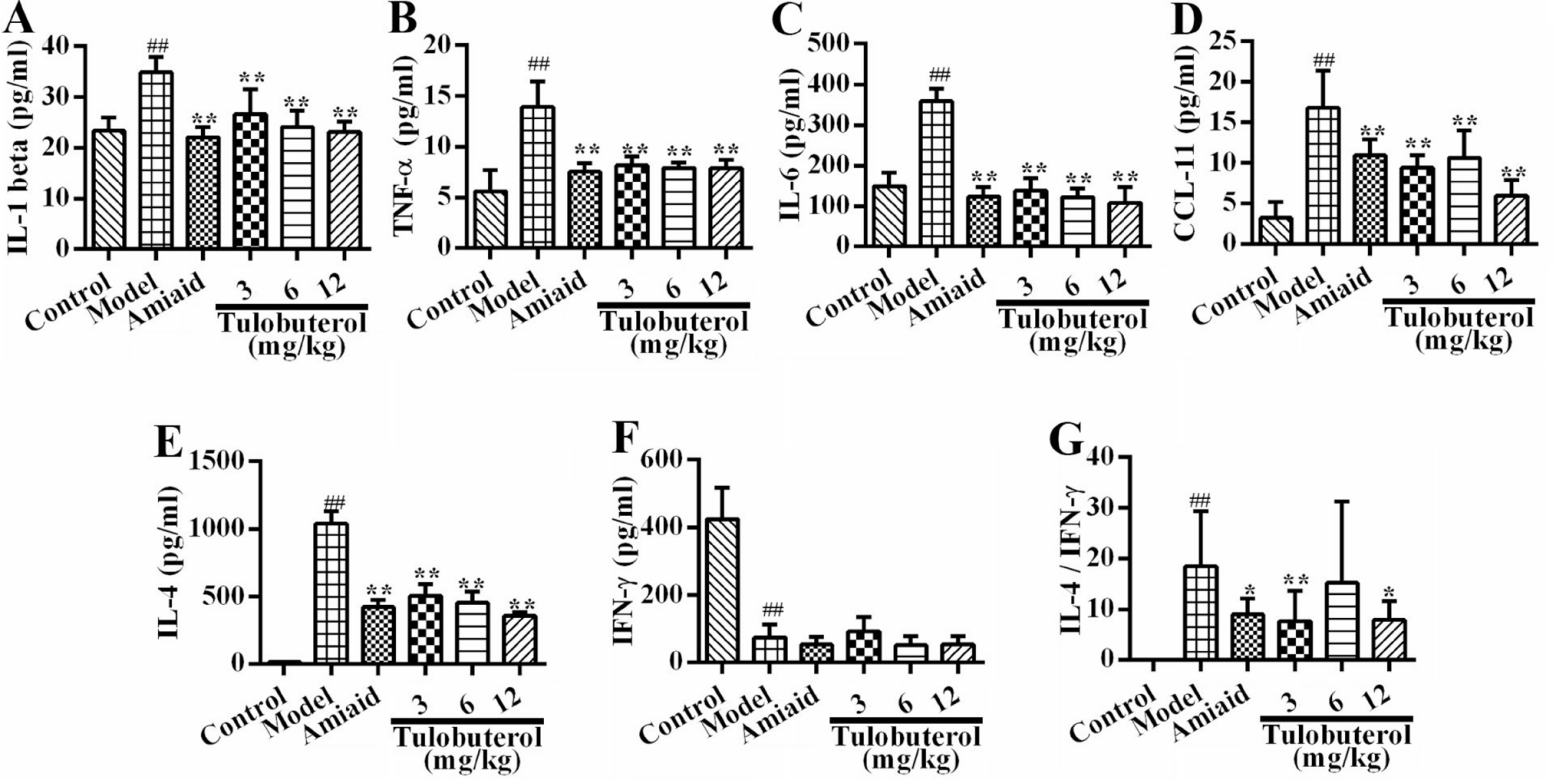
Effects of tulobuterol patch on the levels of inflammatory mediators in BALF (**A**) The level of IL-1β; (**B**) The level of TNF-α; (**C**) The level of IL-6; (**D**) The level of CCL-11; (**E**) The level of IL-4; (**F**) The level of IFN-γ; (**G**) The ratio of IL-4/IFN-γ. Data were expressed as mean ± SD (*n* = 10); ^#^*P* < 0.05 and ^##^*P* < 0.01 *vs.* control group; ^*^P < 0.05 and ^**^P < 0.01 *vs.* model group.

The levels of IL-1β, TNF-α, IL-6, CCL-11, IL-4 and IFN-γ in BALF were assessed by ELISA. Compared with the control group, the levels of IL-1β (Figure 2A), TNF-α (Figure 2B), IL-6 (Figure 2C), CCL-11 (Figure 2D), IL-4 (Figure 2E) in model group were significantly increased, while the level of IFN-γ (Figure 2F) were significantly decreased. Compared with the model group, tulobuterol patch treatment significantly decreased the levels of IL-1β, TNF-α, IL-6, CCL-11 and IL-4 (*p* < 0.01). Importantly, tulobuterol patch treatment significantly reduced the ratio of IL-4/IFN-γ (*p* < 0.05 or *p* < 0.01, Figure 2G), which showed to down-regulate Th2 allergic airway inflammatory phenotypes. Collectively, tulobuterol patch treatment attenuated OVA-dependent inflammatory mediators secretion in BALF, and down-regulated a Th2 allergic inflammatory response.

### Tulobuterol patch attenuated the inflammatory cells infiltration in allergic mice

**Figure 3:**
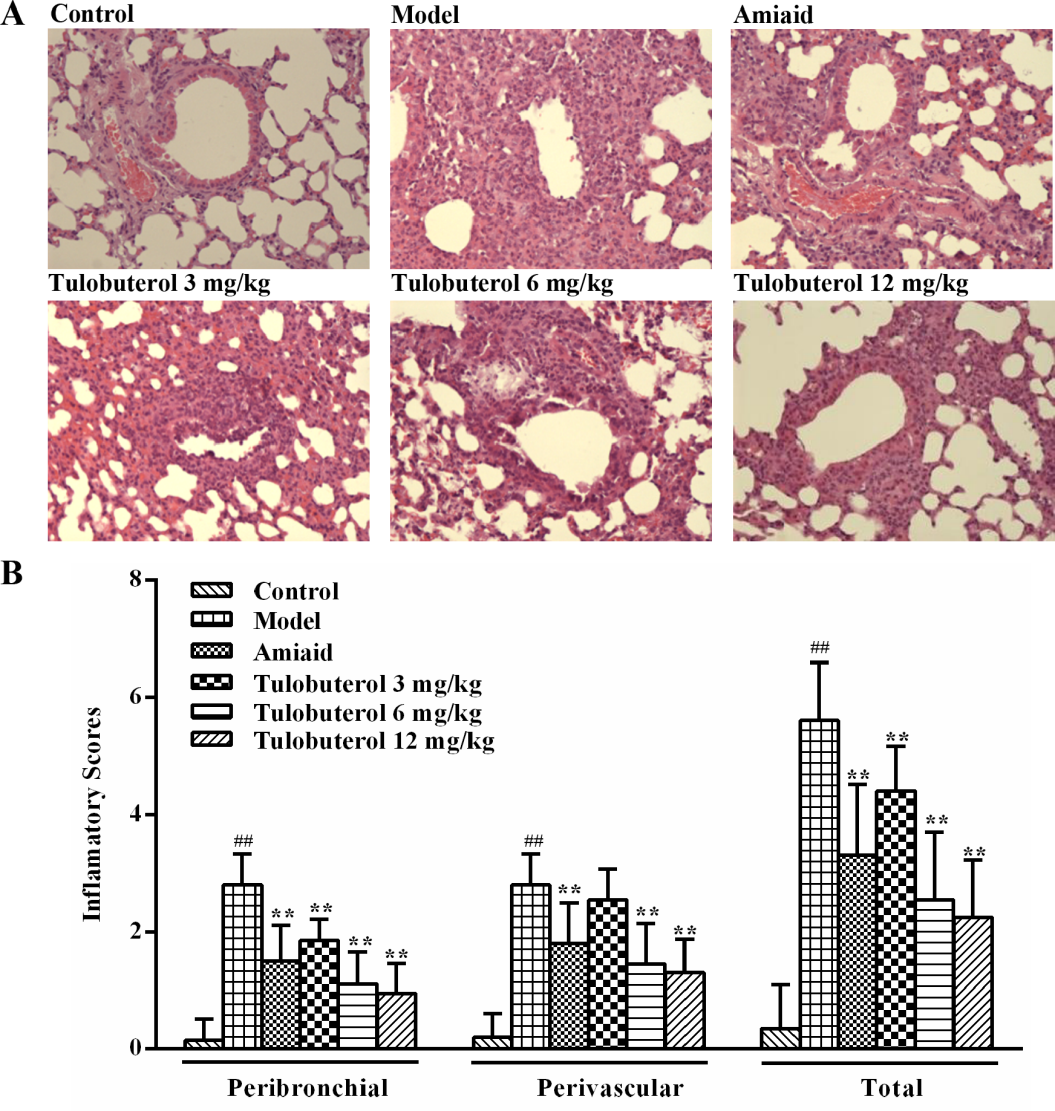
Effects of tulobuterol patch on the inflammatory cells infiltration in allergic mice (**A**) Pathological changes in lung tissue were determined by H&E staining (magnified ×100). (**B**) The scores of inflammatory cells infilitration in H&E stained lung sections. Data were expressed as mean ± SD (*n* = 4); ^#^*P* < 0.05 and ^##^*P* < 0.01 *vs.* control group; ^*^P < 0.05 and ^**^P < 0.01 *vs.* model group.

H&E-staining was used to assess the effect of tulobuterol patch on airway inflammation of the lung sections (Figure 3A). The quantification of peribronchiolar and perivascular inflammatory cells infiltration were determined by using an inflammatory scores, respectively (Figure 3B). Compared to control mice, OVA-induced mice showed a significantly increasing number and marked infiltration of inflammatory cells into perivascular and peribronchial regions. Tulobuterol patch treatment significantly attenuated the inflammatory cells infiltration in both the peribronchial and perivascular regions compared with the model group (*p* < 0.01). Accordingly, compared to control mice, the model mice showed a significantly increase in total inflammatory scores, while tulobuterol patch treatment significantly attenuated the increase in a dose-depedent manner (*p* < 0.01).

### Comparing the anti-inflammatory activities of tulobuterol patch with other two β_2_-agonists in allergic mice

**Figure 4:**
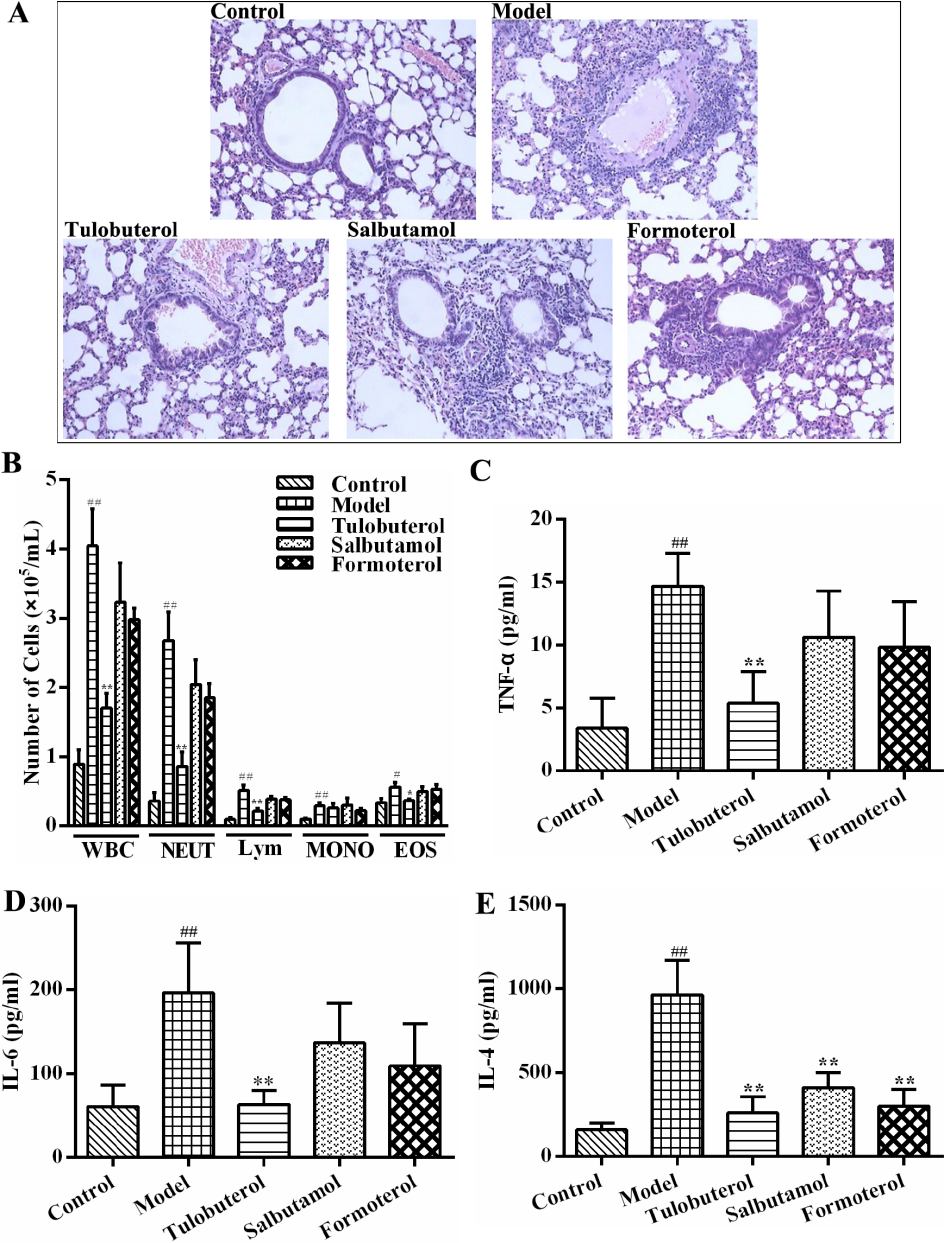
The different anti-inflammatory activities of tulobuterol patch, salbutamol and formoterol in allergic asthma mice (**A**) Pathological changes in lung tissue were determined by H&E staining (magnified ×100, *n* = 3). (**B**) The recruitment of total leukocytes and its differential counts in BALF (*n* = 10); (**C**) The level of TNF-α (*n* = 10); (**D**) The level of IL-6 (*n* = 10); (**E**) The level of IL-4 (*n* = 10). ^#^*P* < 0.05 and ^##^*P* < 0.01 *vs.* control group; ^*^P < 0.05 and ^**^P < 0.01 *vs.* model group.

Two β_2_-agonists, salbutamol and formoterol, were used to observe whether the anti-allergic airway inflammatory activities of tulobuterol patch was different with other β_2_-agonists. As shown in Figure 4A, histopathological examination results showed that the inflammatory cells infiltration in lung peribronchial and perivascular regions were alleviated in all three β_2_-agonists treated mice. Among them, tulobuterol patch treatment exhibited the best effect. The results of leukocyte and its differential counts in BALF also showed that tulobuterol patch treatment significantly decreased the number of total leukocytes, neutrophils, lymphocytes and eosinophils, while no significant decrease was observed with salbutamol and formoterol treatment (Figure 4B). In addition, tulobuterol patch treatment significantly decreased the levels of TNF-α (Figure 4C), IL-6 (Figure 4D) and IL-4 (Figure 4E) in BALF, whereas salbutamol and formoterol treatment only decreased the level of IL-4 significantly. These results exhibited that tulobuterol patch treatment had a more prominently anti-inflammatory potential in allergic asthma besides bronchiectatic activity.

### Tulobuterol patch inhibited the expression and phosphorylation of syk in lung tissues of allergic mice

**Figure 5:**
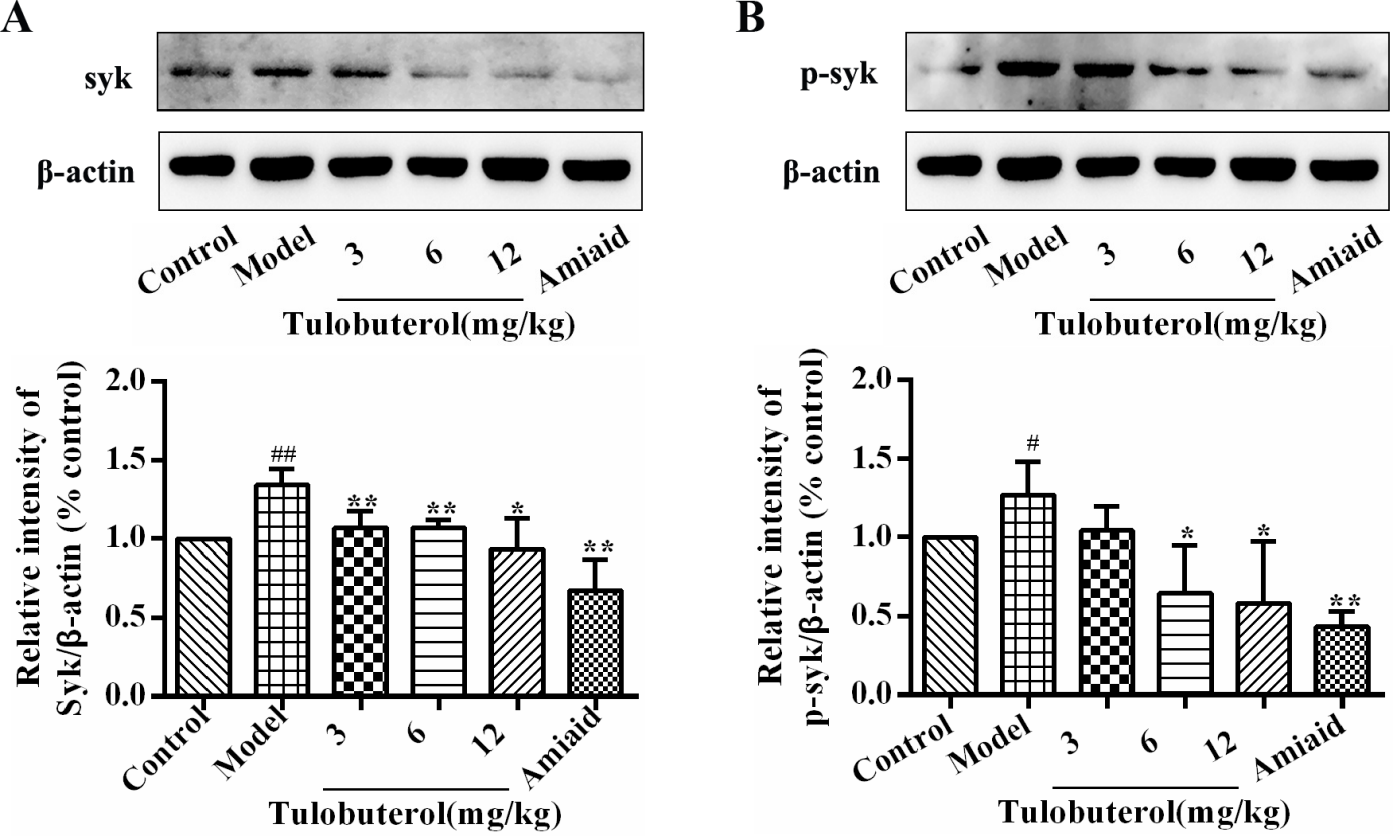
Effects of tulobuterol patch on the expression and phosphorylation of syk in lung tissue (**A**) Western blotting was used to determine the expressions of Syk; (**B**) Western blotting was used to determine the expressions of p-Syk; Data were expressed as mean ± SD (*n* = 3). ^#^*P* < 0.05 and ^##^*P* < 0.01 *vs.* control group; ^*^P < 0.05 and ^**^P < 0.01 *vs.* model group.

Western blotting was employed to determine whether tulobuterol patch had an influence on the expression and phosphorylation of syk in lung tissues. As shown in Figure 5, compared with control group, the levels of syk (Figure 5A) and p-syk (Figure 5B) were significantly increased in model group (*p* < 0.05 or *p* < 0.01), and significantly decreased in a dose-dependent manner with tulobuterol patch treatment (*p* < 0.05 or *p* < 0.01). Collectively, the results suggested that the potential anti-inflammatory mechanism of tulobuterol patch was associated with the syk-mediated pathway.

### The effects of tulobuterol patch on the expression of IκB, NF-κB and p-NF-κB in lung tissues of allergic mice

**Figure 6:**
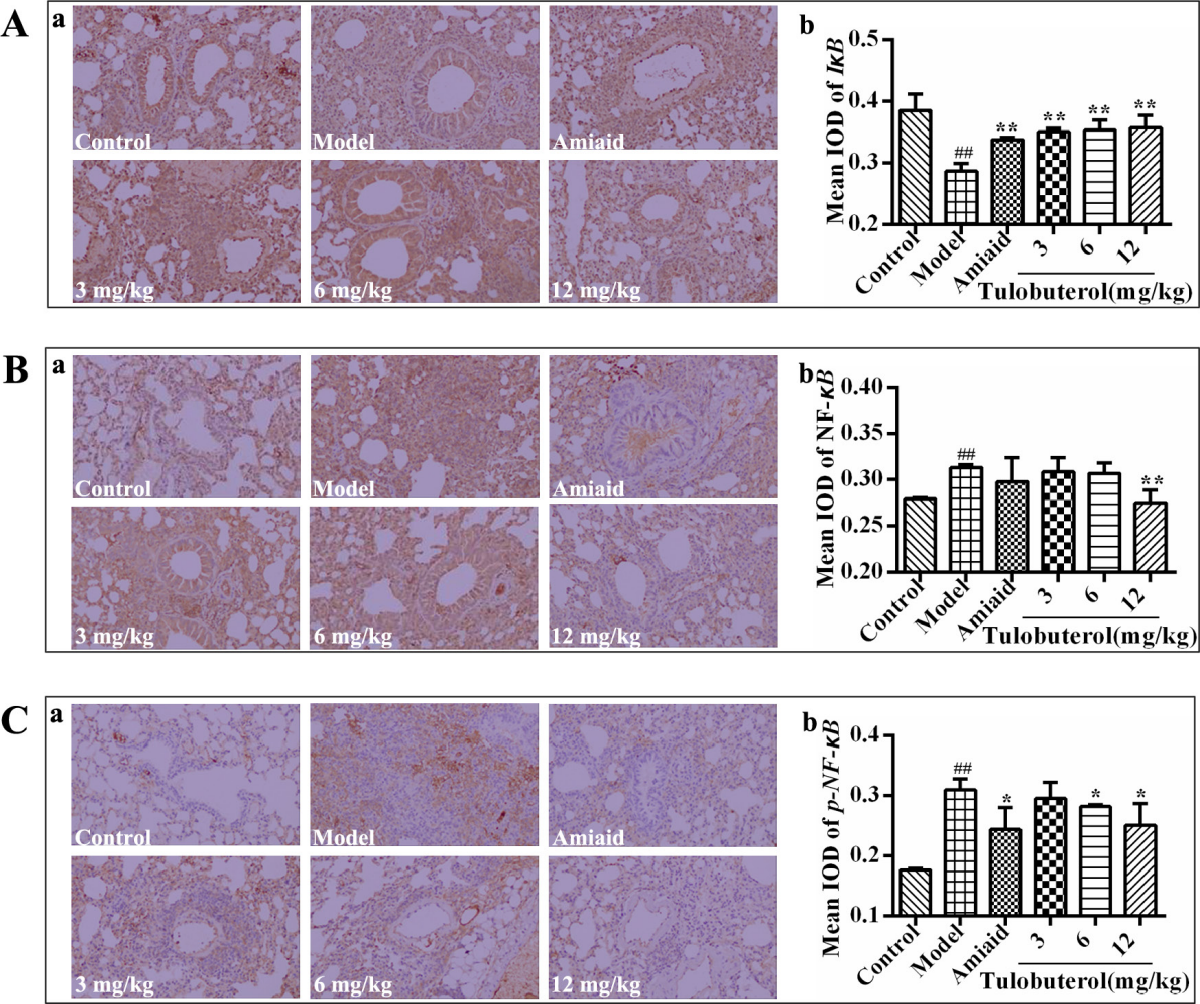
Effects of tulobuterol path on the expressions of IκB, NF-κB, and p-NF-κB detected by immunohistochemistry staining (×200) (**A**) The expression of IκB; (**B**) the expression of NF-κB; (**C**) The expression of p-NF-κB. The mean integrated optical density (IOD) were performed by Image-Pro Plus 6.0 software. Data were expressed as mean ± SD (*n* = 3). ^#^*P* < 0.05 and ^##^*P* < 0.01 *vs.* control group; ^*^P < 0.05 and ^**^P < 0.01 *vs.* model group.

Immunohistochemistry was used to detect the expression of IκB, NF-κB and p-NF-κB in lung tissues. Compared with the control group, the expression of IκB (Figure 6A) was significantly down-regulated, while the expression of NF-κB (Figure 6B) and p-NF-κB (Figure 6C) were significantly increased in model group. Compared with the model group, the expression of IκB was significantly promoted with all dose of tulobuterol patch treatment, the expression of NF-κB was inhibited with 12.0 mg/kg tulobuterol patch treatment, and the expression of p-NF-κB was inhibited with 6.0 mg/kg and 12.0 mg/kg tulobuterol patch treatment, respectively. These results suggested that tulobuterol patch ameliorated the inflammatory responses in lung partly by negatively modulating NF-κB signaling.

## DISCUSSION

β_2_-agonists are the strongest bronchodilators among the standard drugs for bronchial asthma, among which tulobuterol patch has been widely used. Recent studies showed that, by combining with either ICS or leukotriene receptor antagonist, tulobuterol patch enhanced their anti-inflammatory activity in the clinical utility [5, 13]. However, the mechanisms remains not to be conclusively established. Therefore, the anti-inflammatory activities of tulobuterol patch were evaluated, and results showed that treatment of tulobuterol patch induced a markedly reduction in infiltration of inflammatory cells into perivascular and peribronchial in lung of allergic asthma mice.

In allergic asthma, airway inflammation is a Th2 predominant immune response [4], and closely associated with increased production of many inflammatory cytokines and chemokines. IL-4, a Th2 associated cytokine, could inhibit Th1 cell development, promote Th2 cell development, up-regulate B cell costimulatory molecules and lead to an increase in the levels of IgE and IgG1 [14]. Additionally, proinflammatory cytokines, such as TNF-α, IL-1β and IL-6, are found increased in induced sputum from young asthmatic patients [15]. TNF-α facilitates the inflammatory cell immigration into airways, up-regulates adhesion molecules, and activates pro-fibrotic subepithelium [16]. IL-1β promotes recruitment of eosinophils and causes airway responsiveness [17]. IL-6 is a pro-inflammatory cytokine that causes lung injury and fibrosis [18]. CCL11 is secreted by inflammatory epithelial cells and induces eosinophil migration into inflammatory tissue [19]. In the present study, tulobuterol patch treatment significantly prevented the elevated level of IL-4, as well as a decrease in the ratio of IL-4/IFN-γ, suggesting a regulating effect on Th1/Th2 imbalance. Besides, tulobuterol patch treatment reduced TNF-α, IL-1β, IL-6 and CCL11 secretion in BALF, thereby helping to reduce the damage to the respiratory tract. Therefore, it is well demonstrated that tulobuterol patch have a potential of anti-airway inflammation, showing a promising strategy for the development of novel pharmacotherapy in allergic asthma.

In some clinical studies, airway inflammation control was not compromised when patients with ICS were switched to a combination of low dose of ICS with some kinds of β_2_-agonists [5, 13, 20]. In order to compare the anti-airway inflammatory potential of tulobuterol patch with other β_2_-agonists, two commonly used representatives of the short- and long-acting β_2_-agonists, salbutamol and formoterol, were used. Formoterol, a full agonist, have higher intrinsic efficacy than the partial agonist, salbutamol [21, 22]. The partition coefficients (Log P) of tulobuterol, formoterol, and salbutamol are 2.56, 1.06–1.91, 0.34–0.6, respectively [22, 23]. And tulobuterol has the best partition coefficients. Besides, tulobuterol and formoterol are long-acting beta-agonist, which have long duration of action, as opposed to salbutamol [22]. In this study, tulobuterol patch, formoterol and salbutamol were observed to have different magnitudes of anti-inflammatory activity. Tulobuterol patch treatment exhibited the best effect, and had a more prominently anti-inflammatory potential in allergic asthma. Tulobuterol patch treatment significantly alleviated the inflammatory cells infiltration, decreased the leukocyte and its differential count, and decreased the levels of TNF-α, IL-6 and IL-4 in BALF. However, formoterol and salbutamol teatment only significantly decreased the inflammatory cells infiltration in lung peribronchial and perivascular regions and the level of IL-4 in BALF, which were weaker than tulobuterol patch treatment.

Typically, allergic responses of asthma are characterised by hyperproduction of IgE, which bind to and activate the FcεR in macrophages and mast cells [2]. The activated Syk, a cytosolic non-receptor protein tyrosine kinase expressed predominantly in hematopoietic cells, plays a critical role in IgE-induced allergic inflammation, and regulates pro-inflammatory signalings independent of ‘classical’ immmunoreceptor-mediated pathways [11, 12], including initial activation of NF-κB [19]. NF-κB, an important transcription factor presenting in most cell types, is known to be one of the key signaling involved in regulation of a large number of inflammatory mediators including asthma [10, 24] Based on these, in this study, we investigated the potential mechanisms whereby tulobuterol patch regulated the airway inflammation by focusing on the activation of syk/NF-κB signaling. The results showed that the expression and activation of syk were significantly suppressed with tulobuterol patch treatment. Meanwhile, tulobuterol patch treatment also down-regulated the syk downdream signaling NF-κB and p-NF-κB. These results suggest that syk/NF-κB pathway is the important target through which tulobuterol patch mediates the allergic inflammatory response.

In conclusion, the present studies revealed that tulobuterol patch effectively ameliorated airway inflammatory responses in allergic asthma, and its mechanisms, at least partially, via down-regulating syk/NF-κB pathway. These present results contribute for a better understanding of the anti-inflammatory mechanisms of tulobuterol patch, and facilitate the design of pharmacological strategies to optimize its clinical benefit.

## MATERIALS AND METHODS

### Animals care

Male BALB/c mice between 6–8 weeks of age (18–20 g) were purchased from the Experimental Animal Center of Academy of Military Medical Sciences (Beijin,China). The mice were housed in a room on a 12 h light/dark cycle under specific pathogen-free conditions, and had free access to comercial diet and water. All animal experiments were performed in accordance with the Institutional Guidelines for Animal Care and Use of Materia Medica, Chinese Academy of Medical Sciences & Peking Union Medical College.

### OVA-induced allergic asthma in mice and treatment

Mice were randomly divided into six groups: Control, Model, Amiaid (Nitto Denko Corporation, Osaka, Japan) and Tulobuterol patch (Prepared by Prof. Wensheng Zheng, the Institute of Materia Medica of the Chinese Academy of Medical Sciences, Beijing, China). Mice were sensitized with intra peritoneal injection (i.p.) of OVA (Sigma-aldrich, USA) 20 μg plus alum hydroxide (Meihua chemical industry limited company of Shanghai, China) 1 mg in 0.2 ml saline on days 0, 7 and 14, and challenged with intranasal OVA 80 μg in 50 μl saline on days 26–28, then harvested on day 29. The control animals were sensitized and challenged with normal saline in same volume. From day 15 to day 28, mice were smeared with amiaid (6.25 mg/kg) or tulobuterol patch (3, 6, 12 mg/kg, respectly) on skin, once per day.

To compare the anti-inflammatory activities of tulobuterol patch with salbutamol and formoterol, mice were randomly divided into five groups: Control, Model, Tulobuterol patch (6 mg/kg), Salbutamol (orally, 6 mg/kg), and Formoterol (orally, 6 mg/kg). Allergic asthma in mice was induced as described above, and were treated once per day from day 15 to day 28.

### Leukocyte counts in the BALF

Twenty four hours after the last OVA challenge, animals were sacrificed and the BALF were collected by intratracheal instillation of 700 μl of PBS triply. The BALF was centrifuged to collect the whole cells in pellet with 0.5 ml of PBS, the supernatants were carefully removed and stored at –80° C for ELISA. The total leukocyte cell number, neutrophils, lymphocyte, monocyte and eosnophils in BALF were counted in a hematology counter (Beckman Coulter LH 750, USA).

### The measurement of cytokines

Levels of IL-1β, TNF-α, IL-6, CCL-11, IL-4, and IFN-γ in the supernatants of BALF were measured using ELISA kits (Biolegend, San Diego, CA, USA), following the manufacturer instructions.

### Histological analysis

The lung tissue from mice that were not subjected to BALF were fixed in 10% buffered formalin, imbedded in paraffin, cut into 4 μm sections, stained with hematoxylin and eosin (H&E), and analyzed under a light microscope (100×). A 4-point scoring system (grades 0–3) evaluating the degree of the inflammatory cells infiltration (Grade 0: no observable inflammatory cell infiltration; Grade 1: cuff inflammatory cell infiltration next to the veins and bronchus by chance; Grade 2: inflammatory cell infiltration in most of veins and bronchus, inflammatory cells 1–5 layers; Grade 3: inflammatory cell infiltration in most of veins and bronchus, inflammatory cells >5 layers). The evaluation was performed by an observer who was blind to the group.

### Immunohistochemistry

Paraffin-embedded tissues were cut into 5 μm sections, de-paraffinized in xylene, rehydrated through graded alcohol, and rinsed in PBS. Sections were exposed to 3% H_2_O_2_ for 10 minutes to block endogenous peroxidase activity and then placed in EDTA-antigen retrieval. Samples were blocked with sheep serum, incubated with IκB, NF-κB and phosphorylation of NF-κB antibodies (CST, USA) diluted in blocking sera (1:200) at 4° C overnight, incubated with biotinylated secondary antibodies (CST, USA), and then stained using diaminobenzidine chromogen solution at room temperature. Sections were counterstained with haematoxylin, dehydrated and observed under a light microscope. The mean integrated optical density (IOD) was quantified by the Image-Pro Plus 7.0 software.

### Western blotting analysis

For the western blot analysis, the lung tissue were homogenized in ice-cold RIPA lysis buffer (Applygen Technologies Inc, Beijing, China) containing a protease inhibitor cocktail (including protease and phosphatase inhibitor). The lysate was centrifugated at 12000 rpm for 20 min at 4° C, and quantified with BCA method. Equal amount of denatured proteins (30 μg) was separated in a 10% sodium dodecyl sulfate-polyacrylamide gel, electroblotted and transferred onto polyvinglidene difluoride membranes (Millpore, Bedford, MA, USA). The membranes were blocked for 2 h at room temperature in TBST buffer with 5% non-fat milk, and probed with specific primary antibodies for Syk, p-Syk and *β*-actin (CST, USA) at 4° C overnight. After washing 4 times with TBST, the membranes were incubated in the corresponding secondary antibody (HRP-conjugated goat anti-rabbit IgG) for 1 h at room temperature, and the antibody-antigen reactivity was detected using Western Blotting Imaging System (Clinx Science Instruments Co., Ltd, China).

### Statistical analysis

Statistical analyses were performed with SPSS 18.0 (SPSS Inc., Chicago, IL, USA) and statistical significance was set at *P* < 0.05. Data was presented as mean ± SD. As the normality test by Kolmogorov-Smirnov test was passed, data was analyzed by using the Student’s *t-test* for comparison between two groups and one-way ANOVA for multiple groups followed by Fisher’s least significant difference test, otherwise, by using Kruskal-Wallis *H* test.

## Abbreviations

OVA: ovalbumin
ICS: inhaled corticosteroids
Th2: T helper 2
Syk: spleen tyrosine kinase
NF-κB: nuclear factor-κB
BALF: bronchial alveolar lavage fluid
H&E: hematoxylin and eosin
IOD: integrated optical density
